# Developing a pain intensity prediction model using facial expression: A feasibility study with electromyography

**DOI:** 10.1371/journal.pone.0235545

**Published:** 2020-07-09

**Authors:** Riitta Mieronkoski, Elise Syrjälä, Mingzhe Jiang, Amir Rahmani, Tapio Pahikkala, Pasi Liljeberg, Sanna Salanterä

**Affiliations:** 1 Department of Nursing Science, University of Turku, Turku, Finland; 2 Department of Future Technologies, University of Turku, Turku, Finland; 3 Department of Computer Science, University of California, Irvine, California, United States of America; 4 School of Nursing, University of California, Irvine, California, United States of America; 5 Turku University Hospital, Turku, Finland; Swansea University, UNITED KINGDOM

## Abstract

The automatic detection of facial expressions of pain is needed to ensure accurate pain assessment of patients who are unable to self-report pain. To overcome the challenges of automatic systems for determining pain levels based on facial expressions in clinical patient monitoring, a surface electromyography method was tested for feasibility in healthy volunteers. In the current study, two types of experimental gradually increasing pain stimuli were induced in thirty-one healthy volunteers who attended the study. We used a surface electromyography method to measure the activity of five facial muscles to detect facial expressions during pain induction. Statistical tests were used to analyze the continuous electromyography data, and a supervised machine learning was applied for pain intensity prediction model. Muscle activation of corrugator supercilii was most strongly associated with self-reported pain, and the levator labii superioris and orbicularis oculi showed a statistically significant increase in muscle activation when the pain stimulus reached subjects’ self -reported pain thresholds. The two strongest features associated with pain, the waveform length of the corrugator supercilii and levator labii superioris, were selected for a prediction model. The performance of the pain prediction model resulted in a c-index of 0.64. In the study results, the most detectable difference in muscle activity during the pain experience was connected to eyebrow lowering, nose wrinkling and upper lip raising. As the performance of the prediction model remains modest, yet with a statistically significant ordinal classification, we suggest testing with a larger sample size to further explore the variables that affect variation in expressiveness and subjective pain experience.

## Introduction

Pain as a subjective experience is difficult to assess in situations in which patients have no ability to self-report their pain. These situations are common in intensive care units (ICUs), where pain related to critical illnesses, major surgeries and everyday care procedures is common [1,2] and many of the patients are unable to communicate verbally due to mechanical ventilation or sedation. Most behavioral pain assessment scales validated for critically ill patients share an item measuring the facial expressions connected to pain [3], which is found to be the strongest marker associated with pain assessment in non-communicative patients [4,5]. On behavioral pain assessment scales, facial expressions connected to pain are often referred to as grimacing, frowning and wrinkling of the forehead [6–8], scored by observer interpretation according to the intensity of the expression. However, observer-based subjective evaluations often underestimate pain in others [5,9].

According to the study by Arif-Rahu et al. [10], the actions of critically ill patients’ facial muscles during pain are similar to those of healthy volunteers. There is a consensus on four facial actions most associated with pain, forming the core pain expression: brow lowering, nose wrinkling and lip raising, orbit tightening, and eye closure [11,12]. In some studies, the muscles affecting the mouth have also been associated with pain [13,14]. However, facial expressions have been decoded on the level of action units (AUs) more often than a single-muscle basis. The Facial Action Coding System (FACS), a framework for identifying facial expressions by Ekman and Friesen [15], divides facial movements into action units representing individual components of muscle movements. While the FACS has high reliability [16], using the FACS requires specific training, and the scoring is dependent on the subjectivity of the scorer [17].

Advances have been made in developing systems employing facial expressions of pain for automatic pain detection. Computer vision-based pattern recognition has been developed with fair to excellent classification performance [18] using the FACS coding, videotaped material of the BioVid heat pain database [19,20] and the UNBC-McMaster shoulder pain expression archive database [21,22], as well as in clinical situations in post-operative patients [23]. However, monitoring facial expressions using computer vision is challenging when coping with head orientation changes and the interference of medical accessories over a patient’s face, such as tubes or oxygen masks. Most methods are based on coding every frame of a video based on the FACS, which could be a time-consuming process in real-time application [18]. A promising technology used in automatic pattern recognition is surface electromyography (sEMG), a non-invasive technology measuring the electrical activity of superficial muscles with electrodes placed on the skin [24]. The advantage of a sEMG system is the ability to objectively detect subtle facial muscle activity that can be invisible to observers [25]. However, a recent review by Dawes et al. [26] found only one study using the sEMG method to detect facial pain expressions objectively. Furthermore, the suggested correlation between pain intensity and muscle tension has remained unproven.

The current study is a part of the Smart Pain Assessment Tool project, which is intended to develop a clinically useful automatic pain assessment tool for critically ill patients. The main objective of this study was to evaluate the feasibility of the sEMG method for pain detection using two kinds of gradually increasing pain stimuli in healthy subjects. The aim was to examine the facial muscles that can most feasibly be used to detect pain and investigate predictability of pain intensity with machine learning algorithms based on those muscles.

## Methods

### Study subjects

The study was approved by the Ethics Committee of the Hospital District of South West Finland (ETMK:83/1801/2015). Each study subject provided a written informed consent. The study subjects were recruited via advertisements on the university campus and university websites. Thirty-one (15 male and 16 female) healthy volunteers aged 21–51 years (mean age 33 ± 9.0 years) were included in the study. Following the inclusion criteria, all the study subjects had healthy facial skin and no excess facial hair in the areas where the sEMG sensors would be placed. The general health of the study subjects was ensured through oral enquiry. The study exclusion criteria consisted of having chronic or acute illnesses, being pregnant and taking regular medication during or two weeks preceding the study. The data were collected between December 2015 and April 2016.

### Materials and procedures

#### Facial muscles

Pain-related facial descriptors have been investigated in several studies. Facial muscles for the sEMG measurement were chosen compiling previous study findings as shown in Table 1 [17,27,28]. The facial muscles measured in the study were corrugator supercilii, orbicularis oculi, levator labii superioris, zygomaticus major and risorius.

**Table 1:** Adult pain facial expression descriptors, FACS action units and appearance [26], pain-related AUs and muscles [16,27].

Descriptor	Action Unit	Appearance Change	Muscular Basis
Brow lowerer	AU4	The eyebrows are lowered and pulled together, pushing the eyelid down.	Corrugator supercilii
Cheek raiser	AU6	The skin around the temples and cheeks is drawn towards the eyes, narrowing the eye opening.	Orbicularis oculi
Lid tightener	AU7	The eyelids are tightened, narrowing the eye opening.	Orbicularis oculi
Nose wrinkler	AU9	The skin along the sides of the nose is pulled upward and wrinkled.	Levator labii superioris
Upper lip raiser	AU10	The upper lip is drawn up, with the central portion being raised higher than the lower portions.	Levator labii superioris
Lip corner puller	AU12	The lip corner is pulled up diagonally towards the cheekbone.	Zygomaticus major
Mouth stretcher	AU20	The lips are pulled back and sideways, making the mouth look longer.	Risorius
Eye closer	AU43	The eyes are closed, with no apparent tension in the lids.	Orbicularis oculi

#### Electromyography device

For facial muscle activation recognition, the sEMG signal was captured with a multi-purpose biosignal acquisition device that was developed for health monitoring [29]. This device was designed and manufactured by the IoT4Health research group. Version 1.0 of the device used in the study has previously been tested and reported on by Jiang et al. [30]. The multi-purpose device is capable of eight-channel signal acquisition with 24-bit analogue-to-digital resolution. The sample rate is adjustable, and in this study, it was set to 1000 samples per second. The amplitude of the analogue signals were amplified 24 times before digitalization. Each channel was set as a single-ended connection. The muscle activities of selected facial muscle regions were captured with surface electrodes on the right side of the face with monopolar configuration. Additionally, a reference electrode was placed on the bony area behind the ear. The frontalis sEMG channel on the same side was taken as noise reference signal for adaptive noise cancellation.

#### Test procedures

**Fig 1:**
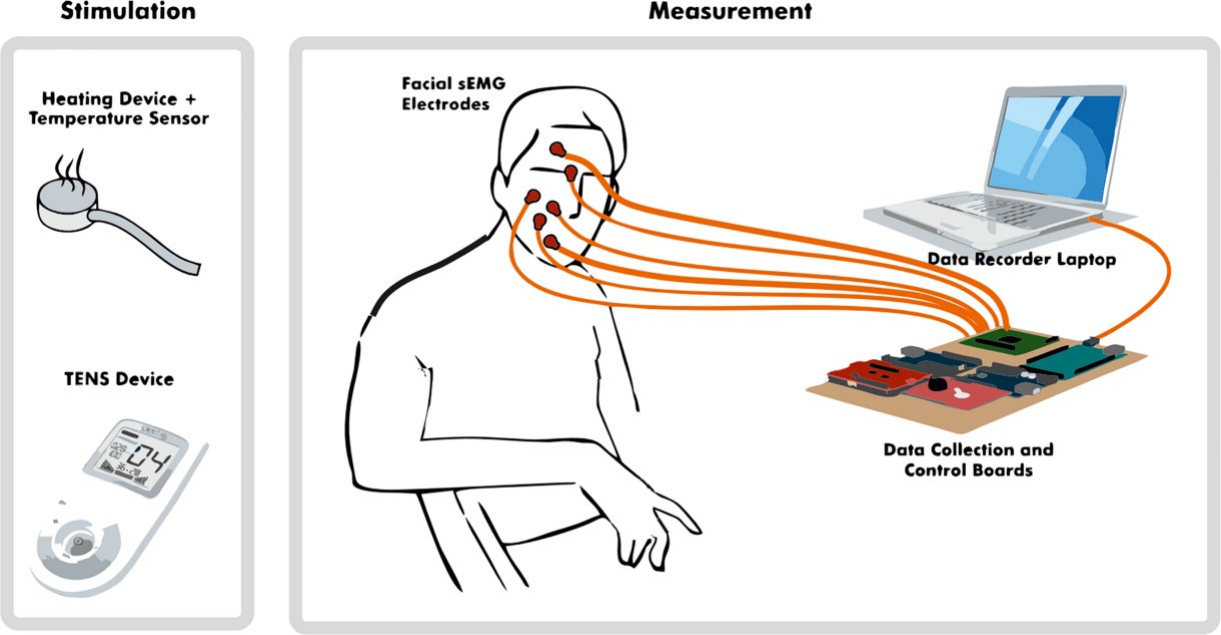
Study stimulation and measurements.

The study was done using a randomized crossover design. The facial sEMG was recorded among other biosignals including heart rate, respiratory rate and skin conductance. The procedures are described and reported with greater detail in Jiang et al. [31]. The study procedures were conducted in a quiet room with a comfortable armchair. A study technician and a study nurse were present during each data-collection session. A non-harmful, slowly increasing pain stimulus was induced with heat and electrical stimulus (shown in Fig 1) on the right of left arms. The subjects were tested four times during each session; two times with each stimulus. The starting test number of the pain induction was randomized to control the order effect. We defined the four tests as 1-left heat, 2-right heat, 3-left electrical pulses and 4-right electrical pulses. A random number between 1 and 4 was generated before all the tests. For example, if the start number was 3, then the order of the test was 3-4-1-2. Heat pain was induced using a round heating element with a diameter of 3 cm in the subject’s inner arm. The heat increased slowly from 30°C at intervals of 0.2–0.3°C per second until reaching 52°C, which is considered a safety limit and the heating process stopped. However, in some cases in which the pain tolerance was not reached before 52°C, the heating element kept warming one to two degrees past the limit with inertia, leading to a mean heat of 52.9°C. A cold pad was applied to prevent any burn marks after each heating session. Electrical stimulus was induced in the fingertip of the ring finger with a transcutaneous electrical nerve stimulation (TENS, Sanitas, Hans Dinlage GmbH, Germany). A pre-installed program with pulse width of 250 μs and the frequency of 100 Hz was selected. The TENS output can be manually increased from level 0 to 50 (peak to peak voltage 2V per level at 500 Ohm) and the levels were increased in every three seconds.

The main motivation of using two pain stimuli in this study was to model generalized experimental pain from physiological signals rather than one specific experimental type of pain. The perception of pain starts from nociceptors, the sensing neurons, sending signals to the spinal cord and brain in response to potentially damaging stimuli. A-Delta and C fibers both carry a certain type of sensory information. The slowing increasing contact heat activates A-Delta fibers, which are responsible for the sensation of a quick and shallow initial pain, and then C fibers, which respond to a deeper, secondary pain. By contrast, electrical stimuli excite nerve fibers directly in the epidermis including the aforementioned nociceptors as well as non-nociceptive fibers [32]. Due to this primary aim, the collected facial sEMG data from all the tests were analyzed altogether.

#### Electromyography data collection

The facial skin under the electrodes was cleaned using cleansing swabs with 70% alcohol before the electrode placement. The pre-gelled H124SG Ag/AgCl sensors (30 mm × 24 mm) were placed on the predetermined facial muscles unilaterally along the right side of the face. The lead wires were attached firmly with tape to avoid disturbing movements. A baseline recording was performed before pain induction began. The pain induction starting place was pre-randomized. Subjects were instructed not to talk during pain induction, to avoid speech-related muscle movement inference. The facial muscle sEMG signals were continuously collected throughout the sessions.

#### Pain intensity assessment

The subjects were instructed to press an alarm signal on two occasions during the pain induction; first, when the sensation was perceived as pain for the first time (pain threshold time point) and second, the pain was intolerable (pain tolerance time point). The pain inducement was stopped within the safety limits even if the pain tolerance was not reached.

### Data analysis

#### Preprocessing and feature extraction

Signal processing and data recomposition were implemented using MATLAB R2014a. The sEMG signal was preprocessed with a 20-Hz Butterworth high-pass filter to remove the movement artefacts and baseline drifts. An adaptive filter was applied for removal of the electrical pulse signals caused by the electrical tests and 50-Hz power line interference. A high-pass filtered frontalis sEMG was used as a noise reference signal in the adaptive noise cancellation. Then, the data were recomposed and down sampled from 1000-Hz signals to 1-Hz features with root mean square (RMS) and waveform length (WL) (Table 2) transformations for each sEMG signal for analysis. The RMS models acted as an amplitude modulated Gaussian random process, whereas the WL represents the sEMG complexity over the time segment [33]. The recomposition produced ten separate features, named with the abbreviation of each muscle (cor = corrugator supercilii, orb = orbicularis oculi, lev = levator labii superioris, zyg = zygomaticus major, and ris = risorius) and feature transformation (rms = root mean square, and wl = waveform length): corrms, corwl, orbrms, orbwl, levrms, levwl, zygrms, zygwl, risrms and riswl (Table 3).

**Table 2:** Data processing.

Order	Processing	Additional Details
	preprocessing	
1	20-Hz Butterworth high-pass filter	
2	Adaptive non-linear noise cancellation	
3	Segmentation and feature extraction: from 1000-Hz signals to 1 Hz signals with root mean square (RMS) and waveform length (WL) transformation	$RMS=\sqrt{\frac{1}{N}\sum_{i=1}^{N}{\left(x_{i}\right)}^{2}}\;WL=\sum_{i=1}^{N-1}\left|x_{i+1}-x_{i}\right|$
	preprocessing	
4	Hampel filtering	The Hampel filter (*K* = 3, *t* = 3) [34], where *K* is the half-window and the outlier threshold is *t* standard deviations
5	Z-score standardization on test level	$z=\frac{\left(x-\mu \right)}{\sigma }$, where μ is the mean, and σ is the standard deviation

**Table 3:** Time mark and period definitions used in the study.

Symbol	Definition
Time Mark	Time
*collected during study*	
t1	Stimulus start
t2	Self-reported pain threshold reached
t3	Self-reported pain tolerance threshold (intolerable) or pain stimulus Safety limit reached/pain induction end
*calculated after study*	
t0	30 seconds before t1
t1split	$t1+\lfloor \frac{t2-t1}{2}\rfloor$ (floor function)	
t2split	$t2+\lfloor \frac{t2-t1}{2}\frac{t3-t2}{2}\rfloor$ (floor function)	
Test period	Time limits	
P0	[t0, t1)	
P1	[t1, t1split]	
P2	(t1split, t2)	
P3	[t2, t2split]	
P4	(t2split, t3]	

Data analysis tasks were performed using Python versions 2.7.14 and 3.6.3 (Anaconda Python for data science). During the feature processing, an additional outlier removal in the form of Hampel filtering was applied to all the data to remove artifact spikes of extremely high amplitude [34] (Table 2). All features were standardized on an individual level for the intra- and inter-person comparison of the sEMG activity between muscles and subjects [35]. The standardization was applied on each subject’s individual test level using z-transformation (zero mean and one standard deviation with a “no pain” period included).

#### Data labelling

**Fig 2:**
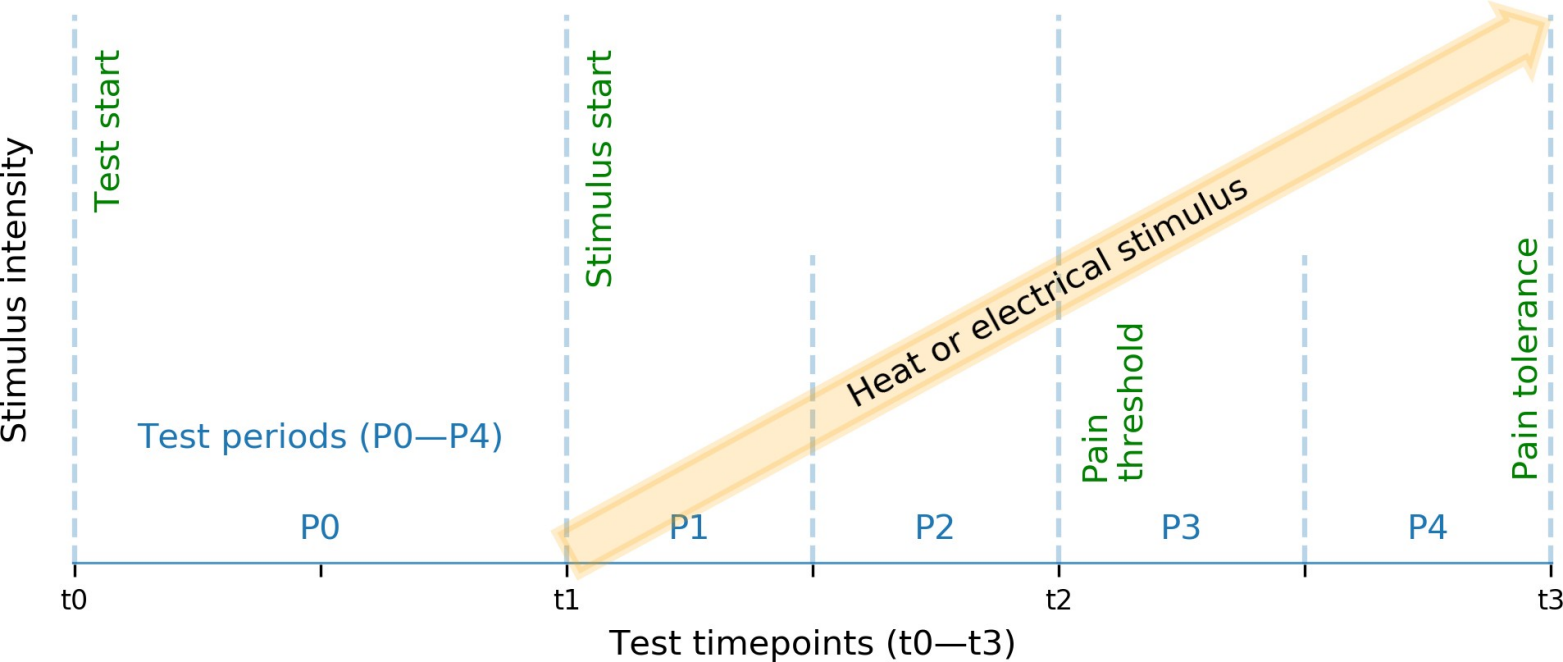
Test schedule. Test periods P0–P4 were defined by test time points stimulus start (t1), subject-reported pain threshold (t2), and pain tolerance (t3).

The test periods defined by stimulus start (t1), subject-reported pain threshold (t2) and pain tolerance (t3) (Fig 2, Table 3) were split linearly to allow more detailed exploration of the level of pain intensity. This resulted in four shorter period labels; P1–P4 (see Table 3). After the data collection, additional test periods P0 were derived by including the collected sEMG signals captured during the 30-second period preceding the start of pain induction.

#### Statistical evaluation of the sEMG features

In the statistical comparison, first, the sEMG features were visually evaluated across the test periods P0–P4. For this, period-specific sEMG feature medians were calculated and plotted. Secondly, a pair-wise statistical comparison of the feature medians was performed. Subject level sEMG feature medians were computed for each period (P1–P4). Finally, inter-correlations between the sEMG features and the test period P1–P4 were determined.

Non-parametric methods were used in the data analysis due to an imbalance of label classes and non-normally distributed sEMG feature values and to avoid linearity assumptions. Thus, we computed Wilcoxon signed-rank statistics [36] for the pairwise comparison of sEMG across the pain periods and Spearman’s rank-correlation for the monotonic relationships.

The k-nearest neighbour (kNN) algorithm was chosen as the machine learning model. The small sample size, unbalanced label classes, non-normally distributed feature values, and the individual differences in the subjects led us to use a simple but efficient non-parametric machine learning method kNN that is able to also capture possible linear, but especially the inherent nonlinearity in the problem.

#### Machine learning modeling

A supervised machine learning model was applied for pain intensity prediction. To study the primary aim, feasibility of pain recognition using sEMG signals, research focused on one method only. The model was constructed using selected sEMG features as the input and periods P1–P4 as the labelled output. The selection process of the sEMG features is described with more detail in the results section. The results from the sEMG feature comparison were used in addition to a table of Spearman’s rank-order correlation coefficients [37] to limit the number of features used in the pain intensity prediction.

The k-nearest neighbour (kNN) algorithm was chosen as the machine learning model. The kNN is a non-parametric method, which performs well with both linear and non-linear patterns. A c-index [38] was used as the performance measure to calculate the concordance between the real ordinal outcomes and model predictions. A c-index is a generalization of the area under the ROC curve (AUC) [39], and values above 0.50 correspond to concordance between the predicted and real categories; values below 0.50 correspond to discordance, and values of 0.50 correspond to random predictions. Unlike the commonly used accuracy, a c-index also gives a reliable measure for unevenly distributed output classes.

In meta learning, the model is designed to automatically select the optimal learning model. The feature selection and hyperparameter k tuning were performed in the prediction model using nested cross-validation. Parameter optimizations were run within an inner loop, while an outer loop was applied for model selection. This method estimated the overall predictability performance without observed data and optimization bias [40]. The within-subject dependence-resulting bias was handled by using leave-subject-out cross-validation [41].

Sometimes, the classifier may produce feasible analysis results without the data itself containing actual patterns due to a small dataset or having too many features [42]. This can be statistically tested using permutation-based p-value. The competence of the meta learning classifier was assessed before the final model evaluation with a permutation-based *p*-value using randomized labels [42] in 1000 permutation tests; the null hypothesis was that the model classifier performance is a result of a random change. This evaluation tested if there is a real connection between the input data and class labels. Leave-subject-out nested cross-validation was applied to the real input values in each test in addition to randomized output classes. A plain non-nested leave-subject-out cross-validation evaluated the final model estimate, with the most suitable parameters produced by the meta learning model.

## Results

In total, 120 tests were included in the analysis; four tests were excluded due to technical problems with the electrical stimulus device. The length of the tests varied between the study subjects and the nature of the pain stimuli. The average duration of a study test involved in the analysis was 110 seconds (SD = 42 seconds). In this study, all the tests were analyzed togetherin alignment with our initial aim to build a general model rather than one specific to a single stimulus.

**Fig 3:**
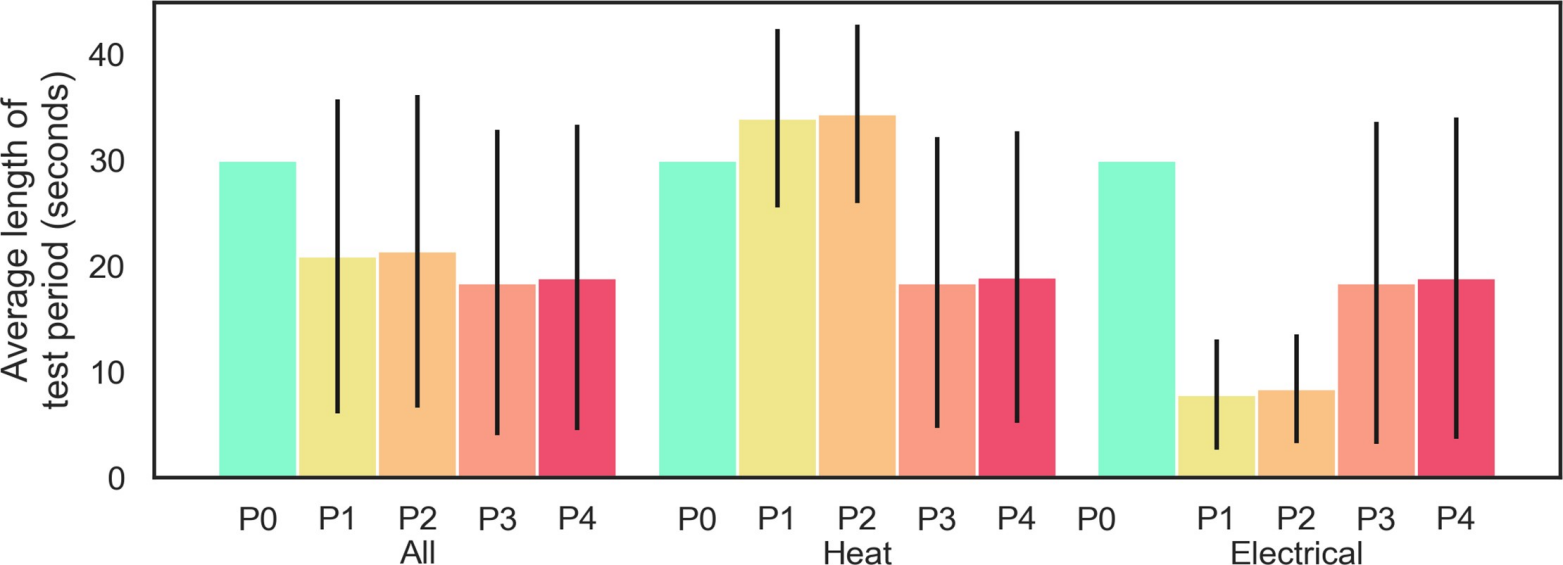
Data distribution across test periods with respect to all tests and then only heat or electrical tests. Bars represent the mean duration of each test period (P0–P4), and the confidence interval shows individual period standard deviations; All–all 120 tests, Heat–only heat stimulus, and Electrical–only electrical stimulus.

Distribution of the self-report-based test period length was almost balanced (Fig 3, ‘All’), especially during the pain induction. A more detailed visual inspection on the pain stimuli level average period length (Fig 3, ‘Heat’, ‘Electrical’) revealed that the heat and electrical stimuli had different threshold period lengths and that the distribution of all sEMG signal distributions were skewed heavily to the right. Therefore, the study data were considered to be non-normally distributed.

**Fig 4:**
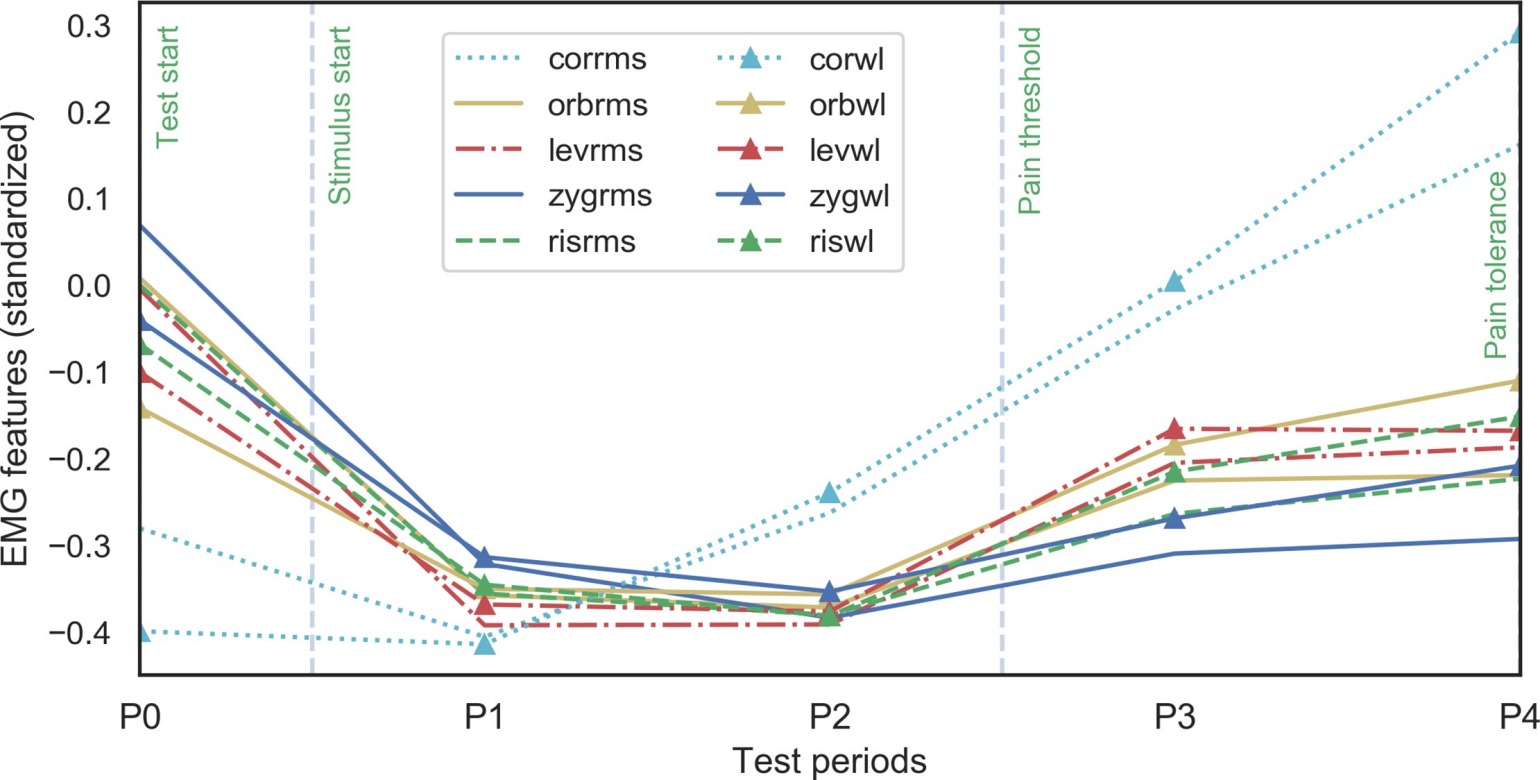
Medians of standardized sEMG features (RMS and WL) across test periods P0–P4. Muscle areas: cor = corrugator supercilii, orb = orbicularis oculi, lev = levator labii superioris, zyg = zygomaticus major, and ris = risorius; feature transformations: rms = root mean square, and wl = waveform length.

The sEMG signal across the test periods was analyzed visually and then statistically. The visual comparison of the standardized sEMG feature medians (see Fig 4) exhibited clear differences between different muscle areas across periods. The corrugator supercilii sEMG-derived RMS and WL feature medians were distinguishable from the other sEMG features. The corrugator muscle activity grew simultaneously with the pain stimulus, whereas the point estimates of the other muscle areas altogether followed similar patterns across study periods P0–P4 from high activation during no pain to low activation at period P1. Eventually, they exhibited slower growth throughout the increasing pain stimulus intensity during the periods P3–P4. The study subjects were not allowed to talk during the pain induction periods. Because this restriction was not present during the later-added no-stimulus period P0, and the visualization showed that period P0 sEMG signals were impacted by talking, all P0 period sEMG signals were excluded from the following analysis.

The statistical analysis over test periods P1–P4 included a pairwise comparison of sEMG features. According to the Wilcoxon signed-rank statistics, corrugator supercilii activity increased across periods. The increase was statistically significant (*p* < 0.05) during all test periods, except the RMS values during period P3 vs. P4. Corrugator features were the only ones that showed a statistically significant difference during the period P1 vs. P2 and P3 vs. P4.

The activity increase of the levator labii superioris was not statistically significant during the test period P1 vs. P2 or the period P3 vs. P4 but showed significant increase in all tests when comparing the periods P1 and P2 before the pain hold vs. periods P3 and P4 after the pain threshold. The pairwise test results of orbicularis oculi activation was similar to levator labii superioris, with the difference in that RMS was not statistically significant when comparing the P1 period and P4 periods.

The change in activation of all five tested muscles in both feature transformations was statistically significant when comparing periods of P2 and P3, representing the time that the subject reported the pain threshold. The p values of the Wilcoxon signed-rank statistics are presented in Table 4.

**Table 4:** Pairwise comparison (Wilcoxon signed-rank, n = 31) across test periods P1–P4 using muscle sEMG feature medians of the individual subjects.

Muscle feature	*p*-value					
	P1 vs. P2	P1 vs. P3	P1 vs. P4	P2 vs. P3	P2 vs. P4	P3 vs. P4
corrms	0.013*	<0.001**	<0.001**	<0.001**	<0.001**	0.088
corwl	0.015*	<0.001**	<0.001**	<0.001**	<0.001**	0.046*
orbrms	0.367	0.018*	0.057	<0.001**	<0.001**	0.422
orbwl	0.422	0.010*	0.008**	<0.001**	<0.001**	0.953
levrms	0.829	0.001**	0.015*	<0.001**	0.001**	0.493
levwl	0.984	0.003**	0.022*	<0.001**	0.001**	0.784
zygrms	0.085	0.290	0.570	<0.001**	0.023*	0.433
zygwl	0.164	0.136	0.583	0.001**	0.024*	0.357
risrms	0.638	0.023*	0.088	<0.001**	0.003**	0.597
riswl	0.597	0.048*	0.071	0.002**	0.004**	0.456

Muscle areas: cor = corrugator supercilii, orb = orbicularis oculi, lev = levator labii superioris, zyg = zygomaticus major, and ris = risorius; feature transformations: rms = root mean square, and wl = waveform length.

*p < 0.05

**p < 0.01

Irrelevant and correlated features were identified using the sEMG signal visual and statistical analysis together with Spearman’s rank correlation coefficient [37] (Table 5). The features selected for the predictive meta learning phase were corrms, corwl, levwl, and orbwl. The overall predictability performance of the meta model produced an average c-index value 0.63. The best performing feature combination in the prediction model included the *features* presenting the waveform lengths of muscles corrugator supercilii and levator labii superioris.

**Table 5:** Inter-correlations (Spearman’s Rho) between the sEMG signals (input), and the period P1–P4.

	corrms	corwl	orbrms	orbwl	levrms	levwl	zygrms	zygwl	risrms	riswl
**periods P1**–**P4**	0.302	0.328	0.149	0.210	0.200	0.218	0.065	0.127	0.153	0.183
	**corrms**	0.871	0.532	0.528	0.547	0.531	0.367	0.401	0.481	0.463
		**corwl**	0.489	0.559	0.488	0.554	0.332	0.437	0.454	0.512
			**orbrms**	0.832	0.798	0.732	0.751	0.668	0.750	0.659
				**orbwl**	0.713	0.803	0.637	0.760	0.693	0.768
					**levrms**	0.834	0.678	0.602	0.746	0.645
						**levwl**	0.616	0.694	0.692	0.747
							**zygrms**	0.759	0.780	0.659
								**zygwl**	0.695	0.807
									**risrms**	0.807

Muscle areas: cor = corrugator supercilii, orb = orbicularis oculi, lev = levator labii superioris, zyg = zygomaticus major, and ris = risorius; feature transformations: rms = root mean square, and wl = waveform length.

The permutation test results of the meta model showed a statistically significant difference (p < 0.01) and therefore rejected the null hypothesis of classifier performance being a result of a random change, which confirmed the significance of the model classifier.

**Fig 5:**
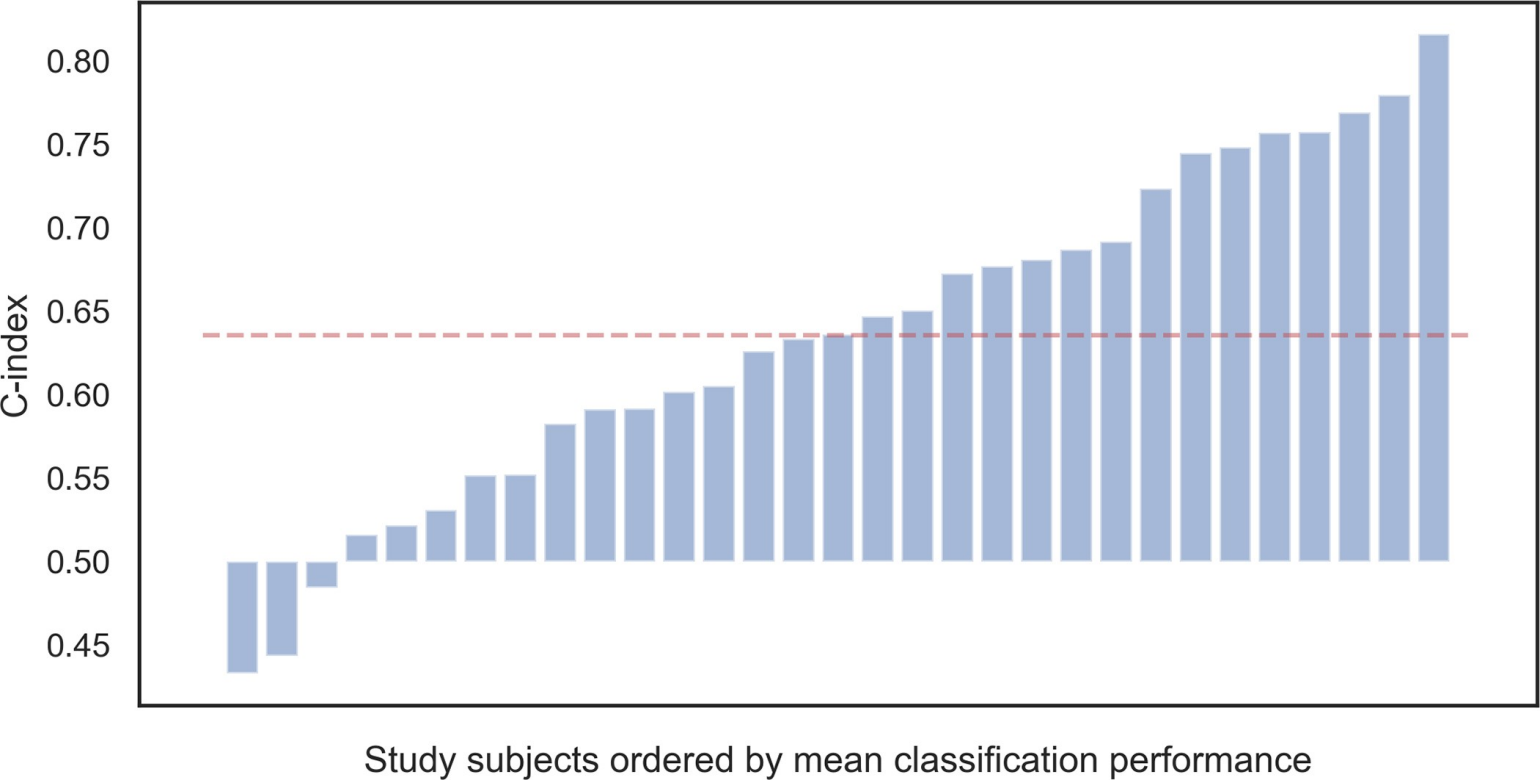
The average subject performance. The average concordance performance (c-index) of the ordinal classification for each subject (ordered by average c-index) compared to the random prediction level (0.5). Red (dashed) line shows the average performance of the final model (0.64).

The final kNN model evaluation with the features corrugator supercilii WL and levator labii superioris WL and the optimized hyperparameter resulted in only moderate prediction of pain intensity (c-index 0.64). Subject level concordance performance varied from random to good (c-index = 0.43, 0.82, respectively). The average subject performance is visualized in Fig 5.

## Discussion

The feasibility of sEMG for pain assessment can be examined from various angles. In this study, we tested the sEMG method measured using a non-commercial multipurpose biosignal acquisition device on healthy subjects to explore which facial muscles are the most feasible for a pain detection and prediction model.

In our results the facial muscles most associated with pain were congruent to the muscle actions described as the core facial pain expression in existing literature [11,12,17]. The corrugator supercilii seemed to be the most feasible muscle area for the recognition of gradually increasing continuous experimental pain in all of our analyses. This is in line with previous findings, which have found corrugator supercilii to be the “muscle of pain” [11,17,43]. The levator labii superioris also reacted strongly to the pain stimuli when the stimulus reached the pain threshold causing cheek raising and nose wrinkling. Our findings are also partly consistent with the study of Wolf et al. [44], in which nine facial muscles were analyzed using the sEMG method during experimental pain induction with a laser system in a sample of 10 male healthy volunteers. They found two muscle groups to be mainly related to pain expression: the orbicularis oculi, as strongest muscle related to eye narrowing, and the mentalis and depressor anguli oris, which cause movements around the mouth. The corrugator supercilii also showed significant results in their analysis as a part of eye narrowing.

The lower perioral muscles around the mouth were not included in our study due to our aim to avoid placing electrodes in impractical positions, considering the care procedures and medical accessories in the ICU. Furthermore, the anatomical differences in facial musculatures and soft tissues form some challenges to the feasibility of sEMG use in clinical care. The medial part of the corrugator supercilii is located just superior to the eyebrow [45], forming an easily located landmark for electrode placement. On the contrary, the upper perioral region muscles located on the cheek are more difficult to distinguish, because the facial muscles are typically very thin and located in layers [46]. Especially risorius cannot be identified in many individuals [47]. Our findings suggest that the risorius might not be a feasible muscle to be used as a predictive measure of pain expression.

Using sEMG signals during the continuously increasing pain stimuli period, the data were classified into four pain intensities. The ordinal classification of the most feasible sEMG signals, the corrugator supercilii and levator labii superioris, resulted in model performance of 0.64. This is better than a random result, but it does not reach a good prediction level. However, for some subjects, the ordinal classification provided a fair pain intensity prediction (eight subjects with c-index > 0.70), whereas some were somewhat randomly ranked (six subjects with the c-index values of 0.43–0.55). This could be a result of the subjects’ differences in facial expressiveness and non-expressiveness [48]. Pain experience is subjective and has variability; therefore, the predictive model could benefit of more items that support the individual differences rather than one-fits-all generalization [49]. With 31 study subjects, a more detailed model was not realizable, but the sample size was reasoned with the feasible nature of the study. Furthermore, in a study design with intentional pain induction, the sample size must be ethically considered. In addition to the subjectivity of the pain experience, pain expression is a diverse non-verbal action to communicate pain to others, involving both voluntary and involuntary actions. It may be influenced by situational differences, but not as easily as the self-report of pain. The influence of the social presence and characteristics of the experimenter may influence the pain tolerance and pain intensity, but the pain threshold seems to be more resistant to external influences [50].

### Limitations

Our study design has weaknesses. Each participant was tested four times with experimental pain, and a carryover effect can occur when multiple interventions are tested on the same study subject [51]. In this study, the carryover effect might have affected the pain experience or the facial pain expression in spite of our attempts to control the carryover effect with randomization. Furthermore, adding a non-painful control intervention that distinguishes expressions unrelated to pain during the study and possibly monitoring a decreasing pain stimulus would have strengthened the design.

Demographic information collected from the subjects consisted of age and sex, as we aimed to have equal numbers of male and female subjects in our sample. These variables were not used in the analysis due to the limited sample. Adding demographic variables related to ethnic backgrounds would have strengthened the insight into the variability of facial actions [52]. Furthermore, the complex phenomenon of facial aging influences changes in the facial bones, soft tissues and skin [53]. Also, muscle contraction amplitude in facial muscle sEMG may be influenced by aging [54], and skinfold thickness may affect signal selectivity [55].We did not test the system of the person’s natural social situation, and the test might have involved contextual factors that affected the results in a study [56]. The participants were aware of the overall study aims to detect various biosignals during pain induction. The study subjects may have applied different emotion regulation strategies during pain inducement that can moderate their pain expression [57]. However, in addition to facial electrodes, the participants wore electrodes on their fingers and in a belt around their chests to detect other biosignals (Reported by Jiang et al. [31]). This might have distracted them from concentrating on the facial expressions only.

The electrodes were applied according the guidelines of the human electromyography study by Fridlund and Cacioppo [58], but it remained unclear whether the recorded muscle activities actually reflected the muscle over which the electrodes were supposed to be fixed or a neighboring muscle. Therefore, when referring to the sEMG measurement of a specific muscle, it would be more appropriate to refer to the muscle group. Also, our choice to use the monopolar configuration may have had an effect on lower selectivity but was reasoned with better usability of the system.

## Conclusions

In conclusion, the feasibility study results show that the muscles that gave the most information in the sEMG measurement during experimental pain were connected to eyebrow-lowering, nose wrinkling and upper lip-raising movements. This is congruent with the previously described core expression of pain. The performance of the prediction model remains modest, but the ordinal classification performance was statistically significant, confirming the pattern between the sEMG features and labels. Pain detection based on facial expressions may need further assurance of subject demographics and other relevant attributes due to the variability in expressiveness of the subjective pain experience.

## Supporting information

S1 Fig Filtered sEMG signals in one test, with extracted waveform length feature. (TIF)

## References

[1] Gélinas C, Chanques G, Puntillo K. In pursuit of pain: Recent advances and future directions in pain assessment in the ICU. Intensive Care Med. 2014 7 6;40(7):1009–14. 10.1007/s00134-014-3299-3 24797682

[2] Chanques G, Sebbane M, Barbotte E, Viel E, Eledjam J-J, Jaber S. A Prospective Study of Pain at Rest: Incidence and Characteristics of an Unrecognized Symptom in Surgical and Trauma versus Medical Intensive Care Unit Patients. Anesthesiology. 2007 11;107(5):858–60. 10.1097/01.anes.0000287211.98642.51 18073576

[3] Pudas-Tähkä SM, Axelin A, Aantaa R, Lund V, Salanterä S. Pain assessment tools for unconscious or sedated intensive care patients: A systematic review. J Adv Nurs. 2009 5;65(5):946–56. 10.1111/j.1365-2648.2008.04947.x 19291192

[4] Severgnini P, Pelosi P, Contino E, Serafinelli E, Novario R, Chiaranda M. Accuracy of Critical Care Pain Observation Tool and Behavioral Pain Scale to assess pain in critically ill conscious and unconscious patients: prospective, observational study. J Intensive Care. 2016 12 7;4(1):68. 27833752 10.1186/s40560-016-0192-x PMC5100216

[5] Ahlers SJGM, van Gulik L, van der Veen AM, van Dongen HPA, Bruins P, Belitser S V, et al Comparison of different pain scoring systems in critically ill patients in a general ICU. Crit Care. 2008;12(1):R15 10.1186/cc6789 18279522 PMC2374638

[6] Gélinas C, Fillion L, Puntillo KA, Viens C, Fortier M. Validation of the critical-care pain observation tool in adult patients. Am J Crit Care. 2006 7 1;15(4):420–7. 16823021

[7] Odhner M, Wegman D, Freeland N, Steinmetz A, Ingersoll GL. Assessing pain control in nonverbal critically ill adults. Dimens Crit Care Nurs. 2003;22(6):260–7. 10.1097/00003465-200311000-00010 14639117

[8] Payen JF, Bru O, Bosson JL, Lagrasta A, Novel E, Deschaux I, et al Assessing pain in critically ill sedated patients by using a behavioral pain scale. Crit Care Med. 2001 12;29(12):2258–63. 10.1097/00003246-200112000-00004 11801819

[9] Prkachin KM, Berzins S, Mercer SR. Encoding and decoding of pain expressions: a judgement study. Pain. 1994;58(2):253–9. 10.1016/0304-3959(94)90206-2 7816493

[10] Arif Rahu M, Grap MJ, Cohn JF, Munro CL, Lyon DE, Sessler CN. Facial expression as an indicator of pain in critically ill intubated adults during endotracheal suctioning. Am J Crit Care. 2013 9 1;22(5):412–22. 10.4037/ajcc2013705 23996421 PMC3913066

[11] Prkachin KM. The consistency of facial expressions of pain: a comparison across modalities. Pain. 1992;51(3):297–306. 10.1016/0304-3959(92)90213-u 1491857

[12] Prkachin KM, Solomon PE. The structure, reliability and validity of pain expression: Evidence from patients with shoulder pain. Pain. 2008 10 15;139(2):267–74. 10.1016/j.pain.2008.04.010 18502049

[13] Craig KD, Patrick CJ. Facial expression during induced pain. J Pers Soc Psychol. 1985 4;48(4):1080–91. 10.1037/0022-3514.48.4.1089 3989673

[14] Kunz M, Lautenbacher S. Improving recognition of pain by calling attention to its various faces. Eur J Pain. 2015 10;19(9):1350–61. 10.1002/ejp.666 25736626

[15] Ekman P, Friesen W V. Facial Action Coding Consulting Palo Alto California: Consulting Psychologists Press; 1978.

[16] Sayette MA, Cohn JF, Wertz JM, Perrott MA, Parrott DJ. A psychometric evaluation of the facial action coding system for assessing spontaneous expression. J Nonverbal Behav. 2001;25(3):167–85.

[17] Prkachin KM. Assessing pain by facial expression: Facial expression as nexus. Vol. 14, Pain Research and Management. ProQuest Central pg; 2009 p. 53–8. 10.1155/2009/542964 PMC2706565 19262917

[18] Sathyanarayana S, Satzoda RK, Sathyanarayana S, Thambipillai S. Vision-based patient monitoring: a comprehensive review of algorithms and technologies. J Ambient Intell Humaniz Comput. 2018 4 26;9(2):225–51.

[19] Walter S, Gruss S, Ehleiter H, Tan J, Traue HC, Crawcour S, et al The biovid heat pain database: Data for the advancement and systematic validation of an automated pain recognition In: 2013 IEEE International Conference on Cybernetics, CYBCONF 2013 2013 p. 128–31.

[20] Werner P, Al-Hamadi A, Niese R, Walter S, Gruss S, Traue HC. Automatic pain recognition from video and biomedical signals In: Proceedings—International Conference on Pattern Recognition. IEEE; 2014 p. 4582–7.

[21] Lucey P, Cohn JF, Prkachin KM, Solomon PE, Chew S, Matthews I. Painful monitoring: Automatic pain monitoring using the UNBC-McMaster shoulder pain expression archive database In: Image and Vision Computing. Elsevier; 2012 p. 197–205.

[22] Lucey P, Cohn JF, Matthews I, Lucey S, Sridharan S, Howlett J, et al Automatically detecting pain in video through facial action units. IEEE Trans Syst Man, Cybern Part B Cybern. 2011; 10.1109/TSMCB.2010.2082525 PMC6942457 21097382

[23] Sikka K, Ahmed AA, Diaz D, Goodwin MS, Craig KD, Bartlett MS, et al Automated assessment of children’s postoperative pain uning computer vision. 2015 7 1;136(1). 10.1542/peds.2015-0029 PMC4485009 26034245

[24] Boxtel A Van. Facial EMG as a tool for inferring affective states. Proc Meas Behav. 2010;2010(August 24–27):104–8.

[25] Wolf K. Measuring facial expression of emotion. Dialogues Clin Neurosci. 2015 12; 17(4):457–62. 26869846 10.31887/DCNS.2015.17.4/kwolf PMC4734883

[26] Dawes TR, Eden-Green B, Rosten C, Giles J, Governo R, Marcelline F, et al Objectively measuring pain using facial expression: is the technology finally ready?. Vol. 8, Pain management. 2018 p. 105–13. 10.2217/pmt-2017-0049 29468939

[27] Ekman P, Rosenberg EL. What the Face Reveals: Basic and Applied Studies of Spontaneous Expression Using the Facial Action Coding System (FACS). What the Face Reveals: Basic and Applied Studies of Spontaneous Expression Using the Facial Action Coding System (FACS). 2012. 1–672 p.

[28] Bartlett MS, Littlewort GC, Frank MG, Lee K. Automatic Decoding of Facial Movements Reveals Deceptive Pain Expressions. Curr Biol. 2014 3 31;24(7):738–43. 10.1016/j.cub.2014.02.009 24656830 PMC4034269

[29] Sarker VK, Jiang M, Gia TN, Anzanpour A, Rahmani AM, Liljeberg P. Portable multipurpose bio-signal acquisition and wireless streaming device for wearables In: 2017 IEEE Sensors Applications Symposium (SAS). IEEE; 2017 p. 1–6.

[30] Jiang M, Gia TN, Anzanpour A, Rahmani A-M, Westerlund T, Salantera S, et al IoT-based remote facial expression monitoring system with sEMG signal. In: 2016 IEEE Sensors Applications Symposium (SAS). IEEE; 2016 p. 1–6.

[31] Jiang M, Mieronkoski R, Syrjälä E, Anzanpour A, Terävä V, Rahmani AM, et al Acute pain intensity monitoring with the classification of multiple physiological parameters. J Clin Monit Comput. 2018 6; 10.1007/s10877-018-0174-8 PMC6499869 29946994

[32] Baumgärtner U, Greffrath W, Treede R-D. Contact heat and cold, mechanical, electrical and chemical stimuli to elicit small fiber-evoked potentials: merits and limitations for basic science and clinical use. Neurophysiol Clin. 2012 10;42(5):267–80. 10.1016/j.neucli.2012.06.002 23040698

[33] Phinyomark A, Phukpattaranont P, Limsakul C. Feature reduction and selection for EMG signal classification. Expert Syst Appl. 2012;39(8):7420–31.

[34] Bhowmik S, Jelfs B, Arjunan SP, Kumar DK. Outlier removal in facial surface electromyography through Hampel filtering technique In: 2017 IEEE Life Sciences Conference (LSC). IEEE; 2017 p. 258–61.

[35] Sousa ASP, Tavares JMRS. Surface electromyographic amplitude normalization methods: A review. Electromyogr New Dev Proced Appl. 2012;

[36] Wilcoxon F. Individual Comparisons of Grouped Data by Ranking Methods Two Industry Problems Caused by Release of DDT. J Econ Entomol. 1946;39(2):269–70. 20983181 10.1093/jee/39.2.269

[37] Spearman C. The Proof and Measurement of Association between Two Things. Am J Psychol. 1904;15(1):72–101. 3322052

[38] Harrell FE, Lee KL, Mark DB. Multivariable Prognostic Models: Issues in Developing Models, Evaluating Assumptions and Adequacy, and Measuring and Reducing Errors. Stat Med. 1996;15:223–49. 10.1002/(SICI)1097-0258(19960229)15:4<361::AID-SIM168>3.0.CO;2-4 8668867

[39] Hanley AJ, McNeil JB. The Meaning and Use of the Area under a Receiver Operating Characteristic (ROC) Curve. Radiology. 1982;143:29–36. 10.1148/radiology.143.1.7063747 7063747

[40] Varma S, Simon R. Bias in error estimation when using cross-validation for model selection. BMC Bioinformatics. 2006;7:1–8. 10.1186/1471-2105-7-1 16504092 PMC1397873

[41] Xu G, Huang JZ. Asymptotic optimality and efficient computation of the leave-subject-out cross-validation. Ann Stat. 2012;40(6):3003–30.

[42] Ojala M, Garriga GC. Permutation Tests for Studying Classifier Performance. J Mach Learn Res. 2010;11:1833–63.

[43] Duchenne C-B. The Mechanism of Human Facial Expression. Cambridge University Press; 1990.

[44] Wolf K, Raedler T, Henke K, Kiefer F, Mass R, Quante M, et al The face of pain—a pilot study to validate the measurement of facial pain expression with an improved electromyogram method. Pain Res Manag. 2005;10(1):15–9. 10.1155/2005/643075 15782243

[45] Isse NG, Elahi MM. The corrugator supercilii muscle revisited. Aesthetic Surg J. 2001 5 1;21(3):209–15. 10.1067/maj.2001.116055 19331895

[46] Cattaneo L, Pavesi G. The facial motor system. Vol. 38, Neuroscience and Biobehavioral Reviews. Pergamon; 2014 p. 135–159. 10.1016/j.neubiorev.2013.11.002 24239732

[47] Pessa JE, Zadoo VP, Adrian EK, Yuan CH, Aydelotte J, Garza JR. Variability of the midfacial muscles: Analysis of 50 hemifacial cadaver dissections. Plast Reconstr Surg. 1998;102(6):1888–93. 10.1097/00006534-199811000-00013 9810983

[48] Werner P, Al-Hamadi A, Walter S. Analysis of facial expressiveness during experimentally induced heat pain In: 2017 7th International Conference on Affective Computing and Intelligent Interaction Workshops and Demos, ACIIW 2017. IEEE; 2018 p. 176–80.

[49] Lopez-Martinez D, Picard R. Multi-task Neural Networks for Personalized Pain Recognition from Physiological Signals. 2017 8;

[50] Kállai I, Barke A, Voss U. The effects of experimenter characteristics on pain reports in women and men. Pain. 2004 11;112(1–2):142–7. 10.1016/j.pain.2004.08.008 15494194

[51] Chiang I-C, Jhangiani RS, Price P. Research Methods in Psychology [Internet]. 2nd Canadi. BCcampus; 2015 Available from: https://opentextbc.ca/researchmethods/chapter/experimental-design/

[52] Chen C, Jack RE. Discovering cultural differences (and similarities) in facial expressions of emotion Vol. 17, Current Opinion in Psychology. Elsevier B.V.; 2017 p. 61–6. 10.1016/j.copsyc.2017.06.010 28950974

[53] Farkas JP, Pessa JE, Hubbard B, Rohrich RJ. The science and theory behind facial aging. Plast Reconstr Surg—Glob Open. 2013;1(1). 10.1097/GOX.0b013e31828ed1da PMC4174174 25289202

[54] Yun S, Son D, Yeo H, Kim S, Kim J, Han K, et al Changes of eyebrow muscle activity with aging: Functional analysis revealed by electromyography. Plast Reconstr Surg. 2014;133(4). 10.1097/PRS.0000000000000052 24378349

[55] De la Barrera EJ, Milner TE. The effects of skinfold thickness on the selectivity of surface EMG. Electroencephalogr Clin Neurophysiol Evoked Potentials. 1994;93(2):91–9. 10.1016/0168-5597(94)90071-x 7512925

[56] Van Bavel JJ, Mende-Siedlecki P, Brady WJ, Reinero DA. Contextual sensitivity in scientific reproducibility. Proc Natl Acad Sci U S A. 2016 6 7;113(23):6454–9. 10.1073/pnas.1521897113 27217556 PMC4988618

[57] Hampton AJD, Hadjistavropoulos T, Gagnon MM, Williams J, Clark D. The effects of emotion regulation strategies on the pain experience. Pain. 2015 5;156(5):868–79. 10.1097/j.pain.0000000000000126 25734999

[58] Fridlund AJ, Cacioppo JT. Guidelines for Human Electromyographic Research. Psychophysiology. 1986 9 1;23(5):567–89. 10.1111/j.1469-8986.1986.tb00676.x 3809364

