# Supplementary figures and images for: Developing a pain intensity prediction model using facial expression: A feasibility study with electromyography
Source: PLoS One. 2020 Jul 9;15(7):e0235545. doi: 10.1371/journal.pone.0235545 (PMC7347182; doi:10.1371/journal.pone.0235545)

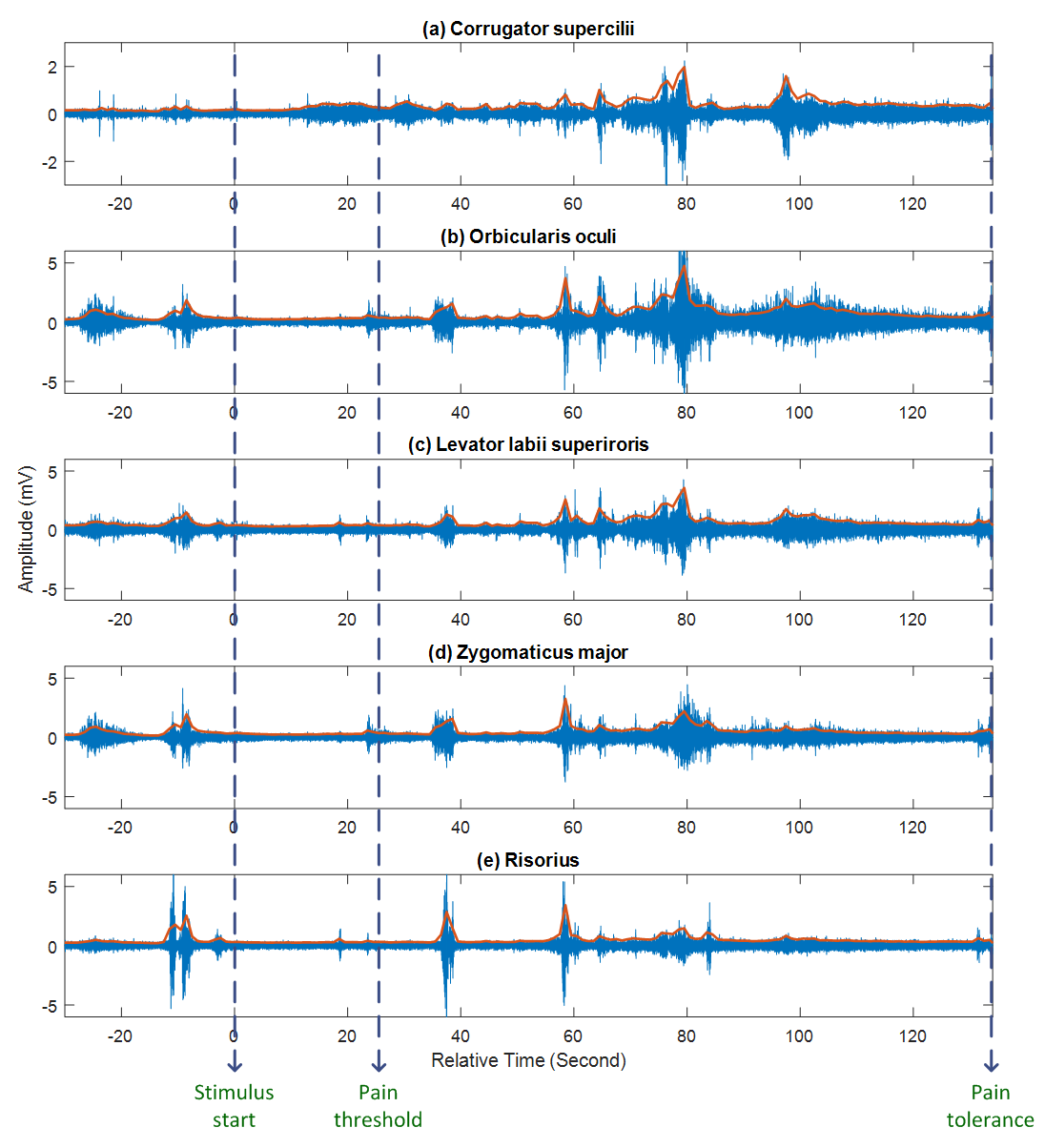

Supplement: S1 Fig — (TIF) [file pone.0235545.s001.tif]
